# Antibacterial Activity of Long-Chain Polyunsaturated Fatty Acids against *Propionibacterium acnes* and *Staphylococcus aureus*

**DOI:** 10.3390/md11114544

**Published:** 2013-11-13

**Authors:** Andrew P. Desbois, Keelan C. Lawlor

**Affiliations:** Marine Biotechnology Research Group, Institute of Aquaculture, School of Natural Sciences, University of Stirling, Stirlingshire, Scotland FK9 4LA, UK; E-Mail: keelanl07@gmail.com

**Keywords:** acne vulgaris, benzoyl peroxide, C20:5*n*-3, combination therapy, docosahexaenoic acid (DHA), natural products, omega-3, skin infection, synergy

## Abstract

New compounds are needed to treat acne and superficial infections caused by *Propionibacterium acnes* and *Staphylococcus aureus* due to the reduced effectiveness of agents used at present. Long-chain polyunsaturated fatty acids (LC-PUFAs) are attracting attention as potential new topical treatments for Gram-positive infections due to their antimicrobial potency and anti-inflammatory properties. This present study aimed to investigate the antimicrobial effects of six LC-PUFAs against *P. acnes* and *S. aureus* to evaluate their potential to treat infections caused by these pathogens. Minimum inhibitory concentrations were determined against *P. acnes* and *S. aureus*, and the LC-PUFAs were found to inhibit bacterial growth at 32–1024 mg/L. Generally, *P. acnes* was more susceptible to the growth inhibitory actions of LC-PUFAs, but these compounds were bactericidal only for *S. aureus*. This is the first report of antibacterial activity attributed to 15-hydroxyeicosapentaenoic acid (15-OHEPA) and 15-hydroxyeicosatrienoic acid (HETrE), while the anti-*P. acnes* effects of the six LC-PUFAs used herein are novel observations. During exposure to the LC-PUFAs, *S. aureus* cells were killed within 15–30 min. Checkerboard assays demonstrated that the LC-PUFAs did not antagonise the antimicrobial potency of clinical agents used presently against *P. acnes* and *S. aureus*. However, importantly, synergistic interactions against *S. aureus* were detected for combinations of benzoyl peroxide with 15-OHEPA, dihomo-γ-linolenic acid (DGLA) and HETrE; and neomycin with 15-OHEPA, DGLA, eicosapentaenoic acid, γ-linolenic acid and HETrE. In conclusion, LC-PUFAs warrant further evaluation as possible new agents to treat skin infections caused by *P. acnes* and *S. aureus*, especially in synergistic combinations with antimicrobial agents already used clinically.

## 1. Introduction

Colonisation of the skin by the anaerobic Gram-positive bacterium *Propionibacterium acnes* is one factor involved in the aetiology of acne vulgaris [1,2]. In addition, colonisation by *Staphylococcus aureus*, an opportunistic Gram-positive pathogen, has been implicated in the pathogenesis of acne [1], and this bacterium can also cause superficial skin infections such as boils, pimples and impetigo [3]. Topical treatment and management of acne relies on the application of benzoyl peroxide (BPO), salicylic acid (SA) and certain antibiotics, while topical *S. aureus* infections are typically treated with fusidic acid (FUS), mupirocin (MUP), neomycin (NEO) and polymyxin B (POL) [4]. However, there has been a decrease in the clinical efficacy of many treatment agents during the last 20 years, perhaps due to the increasing prevalence of drug-resistant *P. acnes* and *S. aureus* strains [5,6,7,8]. Moreover, some of these agents are associated with undesirable side effects such as skin irritation [4], meaning that there is a clinical need to identify alternative compounds with improved therapeutic properties for topical infections caused by *P. acnes* and *S. aureus*.

Fatty acids are attracting attention as potential therapeutic antimicrobial agents due to their potency, broad spectrum of activity and the lack of classical resistance mechanisms against the actions of these compounds [9,10]. In particular, various long-chain polyunsaturated fatty acids (LC-PUFAs), which are found naturally at high levels in many marine organisms [11], have been shown to exert highly potent activity against Gram-positive bacteria, including eicosapentaenoic acid (EPA; C20:5*n*-3) [12,13], docosahexaenoic acid (DHA; C22:6*n*-3) [14,15,16,17], γ-linolenic acid (GLA; C18:3*n*-6) [15,18,19,20,21] and dihomo-γ-linolenic acid (DGLA; C20:3*n*-6) [15]. Furthermore, LC-PUFAs deserve special consideration for topical application because they are often associated with anti-inflammatory properties, which may provide supplementary benefit during therapy [10,22,23]. Thus, the aim of this present study was to investigate the antimicrobial effects of six LC-PUFAs (DGLA, DHA, EPA, GLA, 15-hydroxyeicosatrienoic acid [HETrE], and 15-hydroxyeicosapentaenoic acid [15-OHEPA]) against *P. acnes* and *S. aureus*, to evaluate the potential of these compounds for treating topical infections caused by these pathogens.

## 2. Results

### 2.1. Antibacterial Activity of Six LC-PUFAs against *P. acnes* and *S. aureus*

Minimum inhibitory (MIC) and bactericidal (MBC) concentrations for six LC-PUFAs (DHA, EPA, GLA, DGLA, HETrE and 15-OHEPA) were determined by broth micro-dilution against *P. acnes* NCTC737 and *S. aureus* ATCC43300. In all tests, ethanol and dimethyl sulfoxide (DMSO) carrier solvents had no detectable effect on the growth of these test bacteria. HETrE and DHA were the most effective LC-PUFAs for inhibiting the growth of *P. acnes* (MIC = 32 mg/L) followed by GLA (MIC = 64 mg/L) (Table 1). None of the LC-PUFAs killed *P. acnes* up to the maximum concentration tested (4096 mg/L). Meanwhile, DHA and EPA were the most effective LC-PUFAs against *S. aureus* (MICs = 128 mg/L), but the LC-PUFAs were generally less effective against this bacterium. Indeed, the MIC for each LC-PUFA was up to eight-fold greater against *S. aureus* compared with *P. acnes* (Table 1); however, in contrast to observations for *P. acnes*, each of the six LC-PUFAs was bactericidal against *S. aureus* and MBC values were equal to MIC or 2 × MIC (Table 1). Next, to assess interstrain variability in susceptibility to the LC-PUFAs, MIC_50_ and MBC_50_ values were determined using ten isolates of *S. aureus* from diverse backgrounds, including clinical and laboratory strains and isolates with resistance to methicillin and vancomycin (Table 2). MIC_50_ and MBC_50_ values correspond to the concentrations required to inhibit or kill 50% of the strains tested, respectively. The relative potency of the LC-PUFAs against these *S. aureus* isolates was: DHA > EPA > GLA > HETrE > 15-OHEPA = DGLA (Table 2).

**Table 1:** MICs and MBCs against *P. acnes* NCTC737 and *S. aureus* ATCC43300 for six LC-PUFAs.

Compound	*P. acnes*			*S. aureus*	
	MIC (mg/L)	MBC (mg/L)		MIC (mg/L)	MBC (mg/L)
DGLA	128	>4096		1024	1024
DHA	32	>4096		128	128
EPA	128	>4096		128	256
GLA	64	>4096		512	512
HETrE	32	>4096		256	512
15-OHEPA	128	>4096		512	1024

**Table 2:** MIC_50_ and MBC_50_ values for six LC-PUFAs against ten diverse isolates of *S. aureus*.

Compound	*S. aureus*	
	MIC_50_ (mg/L)	MBC_50_ (mg/L)
DGLA	1024	2048
DHA	128	256
EPA	256	256
GLA	512	512
HETrE	512	1024
15-OHEPA	1024	2048

### 2.2. Killing Kinetics of Six LC-PUFAs against *S. aureus*

**Figure 1:**
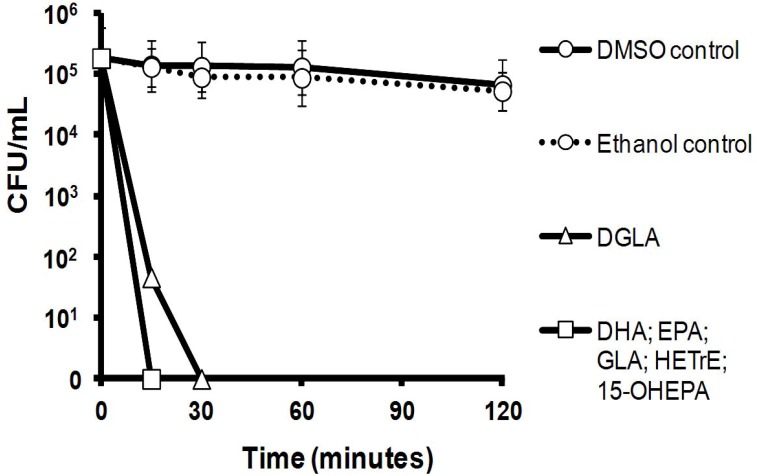
Killing kinetics of six LC-PUFAs against a *S. aureus* ATCC43300 inoculum of ~2.6 × 10^5^ colony forming unit (CFU)/mL in phosphate-buffered saline (PBS) showing that each LC-PUFA killed the bacteria rapidly. *n* = 3; mean ± one standard deviation (not all error bars are visible). Please note that a single kill curve is shown for DHA, EPA, HETrE and 15-OHEPA because the values were identical and the kill curves overlapped exactly.

The killing kinetics of the six LC-PUFAs were determined at 1 × MBC against ~2.6 × 10^5^ colony forming unit (CFU)/mL inoculums of *S. aureus* ATCC43300 prepared in phosphate-buffered saline (PBS). The incubations were performed at 37 °C and there was little change in the viability of cells in the control group that were exposed only to the carrier solvents, ethanol and DMSO. Importantly, the LC-PUFAs killed the *S. aureus* inoculums rapidly and no viable bacteria were detected within 15 min of initial exposure for five of the six LC-PUFAs, with DGLA taking just 30 min to completely kill the inoculum (Figure 1).

### 2.3. Efficacy of Six LC-PUFAs in Combination with Clinical Antimicrobial Agents

Combination therapies are often prescribed during the treatment of acne vulgaris and topical staphylococcal infections [1,2,4] because this approach can improve clinical outcome and may also reduce the opportunity to select for drug-resistant strains [24]. Therefore, to assess whether the LC-PUFAs could be used without reducing the efficacy of agents currently prescribed to treat acne and topical staphylococcal infections, checkerboard tests were performed for each LC-PUFA in combination with six topical antimicrobials. Moreover, these tests enabled us to evaluate whether any of the combinations acted in synergy, as synergistic combinations of antimicrobial agents can deliver enhanced efficacy through greater potency and also reduce undesirable side effects due to the application of lower doses of individual agents [24]. Initially, MICs and MBCs had to be determined against *P. acnes* and *S. aureus* for two antimicrobial agents used to treat acne vulgaris (BPO and SA) and additionally against *S. aureus* for four antibiotics used clinically in the treatment of topical staphylococcal infections (FUS, MUP, NEO and POL) (Table 3). The MIC against *P. acnes* for BPO and SA was 64 mg/L but these agents failed to kill the bacterium up to 4096 mg/L, which was the maximum concentration tested (Table 3). Against *S. aureus*, the MIC and MBC for BPO was 512 mg/L, while the MIC and MBC for SA was 512 mg/L and 1024 mg/L, respectively, meaning that these agents were less potent against *S. aureus* compared with *P. acnes*. FUS and MUP were the most potent clinical agents against *S. aureus* (MICs = 0.25 mg/L), while POL and NEO were less effective (Table 3). Nevertheless, all six clinical antimicrobials exhibited bactericidal activity against *S. aureus* at minimum concentrations up to 64 × MIC (Table 3).

**Table 3:** MICs and MBCs against *P. acnes* NCTC737 and *S. aureus* ATCC43300 for various clinical antimicrobial agents used to treat acne or superficial topical staphylococcal infections. n/d, not determined.

Compound	*P. acnes*			*S. aureus*	
	MIC (mg/L)	MBC (mg/L)		MIC (mg/L)	MBC (mg/L)
BPO	64	>4096		512	512
FUS	n/d	n/d		0.25	8
MUP	n/d	n/d		0.25	16
NEO	n/d	n/d		32	128
POL	n/d	n/d		16	128
SA	64	>4096		1024	2048

Using the pre-determined MIC values, checkerboard assays were performed to detect the interactions between the LC-PUFAs and the clinical antimicrobial drugs against the growth of *P. acnes* and *S. aureus*. The fractional inhibitory concentration (FIC) on duplicate checkerboards was used to calculate a mean FIC for each interaction, and mean FIC ≤ 0.5 was defined as synergy while mean FIC ≥ 4 was defined as antagonism [25]. None of the combinations tested against *P. acnes* gave mean FICs of ≤0.5 or ≥4, indicating no growth inhibitory synergy or antagonism, respectively (Table 4). Against *S. aureus*, none of the combinations gave a mean FIC ≥ 4, indicating no antagonism of efficacy between the LC-PUFAs and the clinical antimicrobial agents (Table 5). Interestingly, several combinations of LC-PUFAs and clinical agents showed antimicrobial synergy against *S. aureus*, including BPO with 15-OHEPA, DGLA and HETrE; and, most notably, NEO with five of the six LC-PUFAs, with the exception being DHA that had an FIC of 0.56 meaning that it only just failed to meet the definition of synergy (Table 5).

**Table 4:** Mean FIC values of interactions between six LC-PUFAs and two topical antimicrobial agents against *P. acnes* NCTC737 showing that none of the combinations were synergistic or antagonistic. *n* = 2; mean ± one standard deviation.

*P. acnes*	DGLA	DHA	EPA	GLA	HETrE	15-OHEPA
BPO	1.50 ± 0.71	1.50 ± 0.71	1.50 ± 0.71	1.50 ± 0.71	0.75 ± 0.00	0.88 ± 0.18
SA	2.00 ± 0.00	1.13 ± 0.53	0.78 ± 0.31	1.50 ± 0.71	1.75 ± 0.35	2.00 ± 0.00

**Table 5:** Mean FIC values of interactions between six LC-PUFAs and six topical antimicrobial agents against *S. aureus* ATCC43300 showing that there was synergy (FIC ≤ 0.5) between BPO with three LC-PUFAs and NEO with five LC-PUFAs. Values in bold indicate synergy; *n* = 2; mean ± one standard deviation.

*S. aureus*	DGLA	DHA	EPA	GLA	HETrE	15-OHEPA
BPO	**0.44 ± 0.09**	2.00 ± 0.00	1.00 ± 0.00	0.56 ± 0.09	**0.50 ± 0.00**	**0.44 ± 0.09**
SA	2.00 ± 0.00	1.25 ± 0.35	0.88 ± 0.18	0.88 ± 0.18	0.88 ± 0.18	1.00 ± 0.00
FUS	0.81 ± 0.27	2.00 ± 0.00	1.13 ± 0.18	1.50 ± 0.71	1.50 ± 0.71	2.00 ± 0.00
MUP	1.50 ± 0.71	1.50 ± 0.00	2.00 ± 0.00	2.25 ± 0.35	2.00 ± 0.00	1.38 ± 0.88
NEO	**0.28 ± 0.13**	0.56 ± 0.09	**0.38 ± 0.00**	**0.19 ± 0.00**	**0.38 ± 0.00**	**0.34 ± 0.04**
POL	1.78 ± 1.72	2.00 ± 0.00	2.00 ± 0.00	1.06 ± 0.62	0.75 ± 0.00	0.94 ± 0.27

## 3. Discussion

New treatments are required for topical infections caused by *P. acnes* and *S. aureus* as the efficacy of many treatments has reduced due to drug resistance and undesirable side effects can also cause problems for patients. In this present study, the antimicrobial effects of six LC-PUFAs were investigated and each compound potently inhibited the growth of *P. acnes* and *S. aureus in vitro*. Moreover, each LC-PUFA was bactericidal against *S. aureus* and caused cell death within just 30 min. Notably, none of the LC-PUFAs antagonised the antimicrobial activity of agents used to treat topical infections caused by these two pathogens; however, various combinations of LC-PUFAs with BPO or NEO acted synergistically to inhibit the growth of *S. aureus*.

Though LC-PUFAs are known to be potently antibacterial [9], to our knowledge this is the first time that 15-OHEPA and HETrE have been shown to exert antimicrobial activities. Furthermore, this is the first study to report that the six LC-PUFAs tested herein are detrimental for the growth of *P. acnes*, though other fatty acids are known to be toxic to this bacterium, including α-linolenic acid (C18:3*n*-3) [26] and lauric acid (C12:0) [27]. Despite LC-PUFAs lacking bactericidal action against *P. acnes*, these compounds did exert similar antibacterial potency as BPO and SA indicating that potency should not prevent LC-PUFAs being considered as alternative agents in acne therapy. The antimicrobial potency of BPO against *P. acnes* in this present study is similar to previous reports, but the lack of bactericidal activity, such as has been reported previously, may stem from methodological differences in quantifying antimicrobial action and the preparation of the inoculum, as well as the use of different growth conditions and bacterial strains [27,28,29]. Despite no indication of *P. acnes* resistance to LC-PUFAs, the probability to select for strains with resistance to the action of these compounds warrants investigation, particularly as a large difference was observed between MIC and MBC values that may increase the opportunity for mutant selection [30].

Confirming previous observations, EPA, DHA and GLA were potently antibacterial against *S. aureus* [18,21,31], but the activity of DGLA against this pathogen is reported here for the first time. Knapp and Melly [32] did show that a C20:3 fatty acid had anti-*S. aureus* activity, but it was not clear which specific fatty acid isomer was used. The LC-PUFAs were similarly potent against a variety of *S. aureus* strains, suggesting that interstrain susceptibility should not present difficulties during future clinical use. The bactericidal action and small differences between MIC and MBC values against *S. aureus* should reduce the chances to select for resistant strains, but this requires experimental confirmation [30].

The LC-PUFAs killed *S. aureus* rapidly with kill times similar to earlier observations for other fatty acids against this bacterium, including caprylic acid (C8:0) [33], lauric acid [34], sapienic acid (C16:1*n*-10) [35,36], oleic acid (C18:1*n*-9) [37], GLA [19] and EPA [38]. Such rapid cell death caused by the LC-PUFAs strongly suggests a cell membrane lytic mechanism of action against *S. aureus*, which is in line with previous studies on Gram-positive species (see review by Desbois and Smith [9]). However, the lack of bactericidal action against *P. acnes* even at high concentrations (4096 mg/L) suggests a different mode of action for LC-PUFAs against this bacterium and growth inhibition could result from disruption at multiple cellular targets [9]. The underlying antimicrobial mechanisms of LC-PUFAs against *P. acnes* merit further investigation.

In checkerboard tests, none of the LC-PUFAs antagonised the efficacy of the topical antimicrobial treatments used against *P. acnes* and *S. aureus*, suggesting that the LC-PUFAs could be added to current treatment regimens without reducing clinical effectiveness. The checkerboards revealed no antimicrobial synergy against *P. acnes* between LC-PUFAs with BPO and SA, even though an earlier study showed that the efficacy of BPO was enhanced in presence of lipid compounds [28]. Against *S. aureus*, three of the six LC-PUFAs acted in synergy with the anti-acne agent BPO, while five of the LC-PUFAs acted synergistically with the aminoglycoside antibiotic NEO. One major benefit of such antimicrobial combinations is the reduced opportunity to select for resistant bacterial strains [24]. Few studies have examined the interactions of fatty acids and LC-PUFAs with clinical antibiotics against bacteria [36,39,40], but the findings in this present study emphasise the potential importance of testing for such synergism as has been highlighted elsewhere [9,13]. At this stage, the nature of the synergy between the LC-PUFAs with BPO or NEO is unknown but one may speculate that they improve the penetration of the clinical agents into the cell because fatty acids can increase cell membrane permeability [41]. The synergy between BPO and certain LC-PUFAs could be exploited to reduce the concentrations of compounds applied to the skin during acne therapy and this could reduce the severity of undesirable side effects. The discovery of synergy between LC-PUFAs and NEO may have uncovered an important new strategy to combat *S. aureus* infections. Synergy has been reported between aminoglycoside antibiotics and a fatty acid-rich oil but few details of the oil composition were given and it was unclear which components were actively involved [42].

Of course, this present study is limited to *in vitro* observations and *in vivo* studies are needed to understand the true potential of applying LC-PUFAs in combination with clinical antimicrobial drugs for treating *P. acnes* and *S. aureus* infections. The prospects for using LC-PUFAs to treat *P. acnes* and *S. aureus* infections appear to be promising because other fatty acids have shown topical efficacy *in vivo* against these pathogens, including lauric acid against *P. acnes* in a murine ear skin infection model [27]; sapienic acid against *S. aureus* in a murine dermatitis model [36]; and oleic acid (C18:1*n*-9) against topical staphylococcal infections in mice [43,44]. Furthermore, 10-undecylenic acid (C11:1*n*-1) is used already as a topical agent for the treatment of fungal infections [45]. Fatty acids are antimicrobial components of the innate immune system found on mammalian skin and the application of exogenous LC-PUFAs during therapy would augment these natural defences [46,47,48,49,50]. Further benefit may be realised because fatty acids can synergise with natural antimicrobial peptides found on human skin [51]. Moreover, reductions in the prescription of conventional antibiotics for topical infections, especially systemic agents, will beneficially reduce the opportunity to select for drug-resistant strains in the non-target skin microbiota [1,7].

## 4. Experimental Section

### 4.1. Reagents and Bacteria

**Figure 2:**
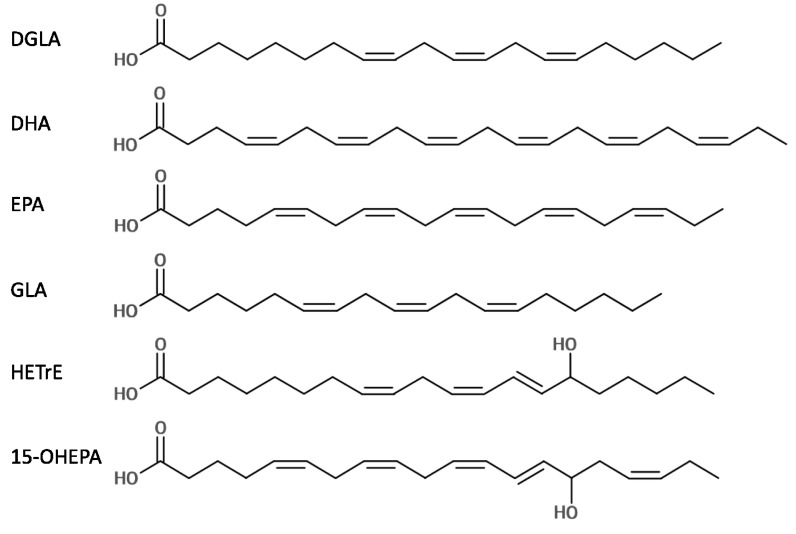
Molecular structures of the six LC-PUFAs used in this present study. All double bonds are in *cis* orientation except for the *n*-7 bonds of 15-HETrE and 15-OHEPA that are in *trans* orientation.

All bacterial culture media and reagents were of the highest purity available and purchased from Sigma-Aldrich Ltd. (Poole, UK) unless stated. The molecular structures of the fatty acids used in this present study are shown in Figure 2. Stock solutions of DGLA (97.9%; Equateq Ltd., Callanish, UK), HETrE (94.3%; Equateq Ltd., Callanish, UK), 15-OHEPA (50 mg/mL; Sapala Organics Pvt. Ltd., Hyderabad, India) and BPO (75%) were prepared in DMSO (≥99.9%) to 50 mg/mL; DHA, EPA and GLA were prepared in ethanol to 100 mg/mL; POL and FUS were prepared in sterile distilled water to 20 mg/mL; while MUP and SA were prepared in ethanol to 50 mg/mL. NEO was purchased as a 10 mg/mL solution in sterile saline. *P. acnes* NCTC737 was purchased from the National Collection of Type Cultures (Porton Down, UK). Ten strains of *S. aureus* were used: two community-acquired meticillin-resistant (MRSA) clinical isolates (CA3 and NRS384), one hospital-acquired MRSA clinical isolate (HA204), two vancomycin-intermediate resistant clinical isolates (5827 and 5836), the reference MRSA strain ATCC43300 (gifted by Dr. Peter Warn, University of Manchester, Manchester, UK), the “laboratory” MRSA strain BB270 and three “laboratory” meticillin-susceptible strains (Newman, SH1000 and RN4220). *S. aureus* strains were sourced as described in Desbois *et al.* [52].

### 4.2. MIC and MBC Determinations

MICs and MBCs were determined for the six LC-PUFA and various clinical antimicrobial agents against *P. acnes* and *S. aureus* by broth micro-dilution according to protocols M07-A8 and M11-A5 published by the Clinical and Laboratory Standards Institute [53,54]. Doubling dilutions of the test compounds were prepared in sterile 96-well plates up to 4096 mg/L. *S. aureus* experiments were performed in Mueller-Hinton broth (MHB), while *P. acnes* studies used supplemented brucella broth (SBB) [54]. Inoculums of *S. aureus* were prepared from late logarithmic phase cultures (~18 h) that had been grown in 5 mL MHB at 37 °C with agitation at 140 rpm, while the *P. acnes* inoculum was prepared in 2–3 mL SBB by re-suspending colonies from supplemented brucella agar (SBA) plates that had been grown anaerobically (10% CO_2_, 10% H_2_, 80% N_2_) for ~72 h at 37 °C (A85 Workstation; Don Whitley Scientific Ltd., Shipley, UK). Absorbance readings at 540 nm and 570 nm of the *P. acnes* and *S. aureus* suspensions were determined respectively, and bacterial inoculums were adjusted to 1 × 10^7^ CFU/mL with fresh medium. Inoculum sizes were confirmed by diluting in phosphate-buffered saline (PBS) and plating on Mueller-Hinton agar (MHA) or SBA. Each well was inoculated with 5 µL of bacterial suspension. Control wells contained media inoculated with bacterial suspension only, while negative control wells contained uninoculated culture medium only. Further inoculated wells controlled for the antimicrobial effects of the greatest concentrations of ethanol and DMSO used in the test wells (4.5% and 9.6%, respectively). Microtitre plates were incubated at 37 °C for 18–20 h (48 h for *P. acnes*), and then the MICs were determined to be the lowest concentrations of each compound that prevented visible bacterial growth. MBCs were determined by plating 50 µL from each well showing no visible growth on to MHA or SBA and incubating these plates at 37 °C for 48 h (96 h for *P. acnes*). The MBCs were the lowest concentrations of each compound that killed ≥99.9% of the initial inoculum. MIC and MBC determinations were performed in duplicate. MIC_50_ and MBC_50_ values were obtained by performing MICs and MBCs against the ten *S. aureus* isolates described above, and then determining the concentration required to inhibit or kill 50% of the strains, respectively.

### 4.3. Kill Kinetics

A *S. aureus* ATCC43300 inoculum was prepared as above, except that PBS was used to adjust the suspension to 1 × 10^7^ CFU/mL. The inoculum was diluted and plated on MHA to provide an accurate indication of CFU/mL. Solutions in PBS of the six LC-PUFAs were prepared at 1 × MBC and 300 µL of each solution was dispensed to separate Eppendorf tubes. Controls contained just ethanol and DMSO diluted in PBS to the greatest concentration of each solvent used (0.5% and 2.1%, respectively). Each tube was inoculated with bacterial suspension (15 µL) and incubated statically at 37 °C. At 15 min, 30 min, 1 h and 2 h, 10 µL from each tube was plated on MHA in duplicate. Plates were incubated at 37 °C for 24 h and then CFU were quantified. The experiment was performed in triplicate.

### 4.4. Checkerboard Tests

Checkerboard assays were performed according to the standard protocol published by the American Society for Microbiology [55] to investigate the antimicrobial interactions between the six LC-PUFAs and two anti-acne agents (BPO and SA) against *P. acnes*. Further checkerboards were performed against *S. aureus* for BPO, SA, FUS, MUP, NEO and POL with these agents being assessed separately against each of the six LC-PUFAs. Doubling dilutions of each compound were prepared in MHB or SBB and dispensed to 96-well plates in the standard checkerboard pattern. Concentrations of each compound ranged from ≤1/16 × MIC to ≥4 × MIC. Control wells were dispensed with medium supplemented with the greatest concentration of solvent used in any well (4.5% ethanol and/or 4.5% DMSO), while negative control wells contained just medium. Inoculums were prepared to 1 × 10^7^ CFU/mL as above. Each well (except for negative controls) was inoculated with 5 µL of bacterial suspension. Plates were incubated as for MIC determinations above and then the presence of bacterial growth was determined for each well according to the clarity of the medium and/or the presence of suspended colonies. The FIC for each well was determined for each interaction and the FICs from duplicate checkerboard assays were used to calculate a mean value that permitted interpretation of the nature of interaction between each combination of compounds.

## 5. Conclusions

There is a need for alternative agents to treat acne and superficial skin infections caused by *P. acnes* and *S. aureus* due to drug-resistance and undesirable side effects associated with certain clinical agents used at present. LC-PUFAs have attracted attention as new agents against these pathogens due to their potent antimicrobial actions and potential anti-inflammatory properties. This present study demonstrates that these natural compounds deserve further evaluation for the treatment of infections caused by *P. acnes* and *S. aureus*, and LC-PUFAs could be applied in combination with some currently available treatments to enhance therapeutic efficacy.

## References

[1] Bojar R.A., Holland K.T. (2004). Acne and *Propionibacterium acnes*. Clin. Dermatol..

[2] Harper J.C. (2004). An update on the pathogenesis and management of acne vulgaris. J. Am. Acad. Dermatol..

[3] Motswaledi M.H. (2011). Superficial skin infections and the use of topical and systemic antibiotics in general practice. S. Afr. Fam. Pract..

[4] Joint Formulary Committee (2011). British National Formulary.

[5] Upton A., Lang S., Heffernan H. (2003). Mupirocin and *Staphylococcus aureus*: A paradigm of emerging antibiotic resistance. J. Antimicrob. Chemother..

[6] El-Zimaity D., Kearns A.M., Dawson S.J., Price S., Harrison G.A.J. (2004). Survey, characterisation and susceptibility to fusidic acid of *Staphylococcus aureus* in the Carmarthen area. J. Antimicrob. Chemother..

[7] Moon S.H., Roh H.S., Kim Y.H., Kim J.E., Ko J.Y., Ro Y.S. (2012). Antibiotic resistance of microbial strains isolated from Korean acne patients. J. Dermatol..

[8] Simonart T., Dramaix M. (2005). Treatment of acne with topical antibiotics: Lessons from clinical studies. Br. J. Dermatol..

[9] Desbois A.P., Smith V.J. (2010). Antibacterial free fatty acids: activities, mechanisms of action and biotechnological potential. Appl. Microbiol. Biotechnol..

[10] Desbois A.P. (2012). Potential applications of antimicrobial fatty acids in medicine, agriculture and other industries. Recent Pat. Antiinfect. Drug Discov..

[11] Berge J.P., Barnathan G. (2005). Fatty acids from lipids of marine organisms: molecular biodiversity, roles as biomarkers, biologically active compounds, and economical aspects. Adv. Biochem. Eng. Biotechnol..

[12] Desbois A.P., Mearns-Spragg A., Smith V.J. (2009). A fatty acid from the diatom *Phaeodactylum tricornutum* is antibacterial against diverse bacteria including multi-resistant *Staphylococcus aureus* (MRSA). Mar. Biotechnol..

[13] Desbois A.P., Kim S.-K. (2013). Antimicrobial properties of eicosapentaenoic acid (C20: 5*n*-3). Marine Microbiology: Bioactive Compounds and Biotechnological Applications.

[14] Coonrod J.D. (1987). Rôle of surfactant free fatty acids in antimicrobial defences. Eur. J. Respir. Dis..

[15] Feldlaufer M.F., Knox D.A., Lusby W.R., Shimanuki H. (1993). Antimicrobial activity of fatty acids against *Bacillus larvae*, the causative agent of American foulbrood disease. Apidologie.

[16] Maia M.R.G., Chaudhary L.C., Figueres L., Wallace R.J. (2007). Metabolism of polyunsaturated fatty acids and their toxicity to the microflora of the rumen. Antonie Van Leeuwenhoek.

[17] Huang C.B., Ebersole J.L. (2010). A novel bioactivity of omega-3 polyunsaturated fatty acids and their ester derivatives. Mol. Oral Microbiol..

[18] Butcher G.W., King G., Dyke K.G.H. (1976). Sensitivity of *Staphylococcus aureus* to unsaturated fatty acids. J. Gen. Microbiol..

[19] Asthana R.K., Srivastava A., Kayastha A.M., Nath G., Singh S.P. (2006). Antibacterial potential of γ-linolenic acid from *Fischerella* sp. colonising Neem tree bark. World J. Microbiol. Biotechnol..

[20] Huang C.B., George B., Ebersole J.L. (2010). Antimicrobial activity of *n*-6, *n*-7 and *n*-9 fatty acids and their esters for oral microorganisms. Arch. Oral Biol..

[21] Zhang H., Zhang L., Peng L., Dong X., Wu D., Wu V.C., Feng F. (2012). Quantitative structure-activity relationships of antimicrobial fatty acids and derivatives against *Staphylococcus aureus*. J. Zhejiang Univ. Sci. B.

[22] Kristmundsdóttir T., Skúlason S., Thormar H. (2011). Lipids as active ingredients in pharmaceuticals, cosmetics and health foods. Lipids and Essential Oils as Antimicrobial Agents.

[23] Mullen A., Loscher C.E., Roche H.M. (2010). Anti-inflammatory effects of EPA and DHA are dependent upon time and dose-response elements associated with LPS stimulation in THP-1-derived macrophages. J. Nutr. Biochem..

[24] Kalan L., Wright G.D. (2011). Antibiotic adjuvants: multicomponent anti-infective strategies. Expert Rev. Mol. Med..

[25] Odds F.C. (2003). Synergy, antagonism, and what the chequerboard puts between them. J. Antimicrob. Chemother..

[26] Ko H.L., Heczko P.B., Pulverer G. (1978). Differential susceptibility of *Propionibacterium acnes*, *P. granulosum* and *P. avidum* to free fatty acids. J. Invest. Dermatol..

[27] Nakatsuji T., Kao M.C., Fang J.-Y., Zouboulis C.C., Zhang L., Gallo R.L., Huang C.M. (2009). Antimicrobial property of lauric acid against *Propionibacterium acnes*: its therapeutic potential for inflammatory acne vulgaris. J. Invest. Dermatol..

[28] Decker L.C., Deuel D.M., Sedlock D.M. (1989). Role of lipids in augmenting the antibacterialk activity of benzoyl peroxide against *Propionibacterium acnes*. Antimicrob. Agents Chemother..

[29] Pannu J., McCarthy A., Martin A., Hamouda T., Ciotti S., Ma L., Sutcliffe J., Baker J.R. (2011). *In vitro* antibacterial activity of NB-003 against *Propionibacterium acnes*. Antimicrob. Agents Chemother..

[30] Blondeau J.M. (2009). New concepts in antimicrobial susceptibility testing: the mutant prevention concentration and mutant selection window approach. Vet. Dermatol..

[31] Shin S.Y., Bajpai V.K., Kim H.R., Kang S.C. (2007). Antibacterial activity of bioconverted eicosapentaenoic (EPA) and docosahexaenoic acid (DHA) against foodborne pathogenic bacteria. Int. J. Food Microbiol..

[32] Knapp H.R., Melly M.A. (1986). Bactericidal effects of polyunsaturated fatty acids. J. Infect. Dis..

[33] Nair M.K.M., Joy J., Vasudevan P., Hinckley L., Hoagland T.A., Venkitanarayanan K.S. (2005). Antibacterial effect of caprylic acid and monocaprylin on major bacterial mastitis pathogens. J. Dairy Sci..

[34] Bergsson G., Arnfinnsson J., Steingrímsson Ó., Thormar H. (2001). Killing of Gram-positive cocci by fatty acids and monoglycerides. APMIS.

[35] Wille J.J., Kydonieus A. (2003). Palmitoleic acid isomer (C16:1Δ6) in human skin sebum is effective against Gram-positive bacteria. Skin Pharmacol. Appl. Skin Physiol..

[36] Clarke S.R., Mohamed R., Bian L., Routh A.F., Kokai-Kun J.F., Mond J.J., Tarkowski A., Foster S.J. (2007). The *Staphylococcus aureus* surface protein isdA mediates resistance to innate defences of human skin. Cell Host Microb..

[37] Chamberlain N.R., Mehrtens B.G., Xiong Z., Kapral F.A., Boardman J.L., Rearick J.I. (1991). Correlation of carotenoid production, decreased membrane fluidity, and resistance to oleic acid killing in *Staphylococcus* aureus 18Z. Infect. Immun..

[38] Shin S.Y., Bajpai V.K., Kim H.R., Kang S.C. (2007). Antibacterial activity of eicosapentaenoic acid (EPA) against foodborne and food spoilage microorganisms. LWT.

[39] Giamarellos-Bourboulis E.J., Grecka P., Dionyssiou-Asteriou A., Giamarellou H. (2000). Impact of *n*-6 polyunsaturated fatty acids on growth of multidrug-resistant *Pseudomonas aeruginosa*: Interactions with amikacin and ceftazidime. Antimicrob. Agents Chemother..

[40] Kitahara T., Aoyama Y., Hirakata Y., Kamihira S., Kohno S., Ichikawa N., Nakashima M., Sasaki H., Higuchi S. (2006). *In vitro* activity of lauric acid or myristylamine in combination with six antimicrobial agents against methicillin-resistant *Staphylococcus aureus* (MRSA). Int. J. Antimicrob. Agents.

[41] Kravchenko I.A., Golovenko N.Y., Larionov V.B., Aleksandrova A.I., Ovcharenko N.V. (2003). Effect of lauric acid on transdermal penetration of phenazepam *in vivo*. Bull. Exp. Biol. Med..

[42] Saraiva R.A., Matias E.F.F., Coutinho H.D.M., Costa J.G.M., Souza H.H.F., Fernandes C.N., Rocha J.B.T., Menezes I.R.A. (2011). Synergistic action between *Caryocar coriaceum* Wittm. fixed oil with aminoglycosides *in vitro*. Eur. J. Lipid Sci. Technol..

[43] Chen C.-H., Wang Y., Nakatsuji T., Liu Y.-T., Zouboulis C.C., Gallo R.L., Zhang L., Hsieh M.F., Huang C.M. (2011). An innate bactericidal oleic acid affective against skin infection of methicillin-resistant *Staphylococcus aureus*: A therapy concordant with evolutionary medicine. J. Microbiol. Biotechnol..

[44] Huang C.-M., Chen C.-H., Pornpattananangkul D., Zhang L., Chan M., Hsieh M.-F., Zhang L. (2011). Eradication of drug resistant *Staphylococcus aureus* by liposomal oleic acids. Biomaterials.

[45] Hart R., Bell-Syer S.E.M., Crawford F., Torgerson D.J., Young P., Russell I. (1999). Systematic review of topical treatments for fungal infections of the skin and nails of the feet. BMJ.

[46] Georgel P., Crozat K., Lauth X., Makrantonaki E., Seltmann H., Sovath S., Hoebe K., Du X., Rutschmann S., Jiang Z., Bigby T., Nizet V., Zouboulis C.C., Beutler B. (2005). A toll-like receptor 2-responsive lipid effector pathway protects mammals against skin infections with Gram-positive bacteria. Infect. Immun..

[47] Drake D.R., Brogden K.A., Dawson D.V., Wertz P.W. (2008). Antimicrobial lipids at the skin surface. J. Lipid Res..

[48] Brogden N.K., Mehalick L., Fischer C.L., Wertz P.W., Brogden K.A. (2012). The emerging role of peptides and lipids as antimicrobial epidermal barriers and modulators of local inflammation. Skin Pharmacol. Physiol..

[49] Thormar H., Hilmarsson H. (2007). The role of microbicidal lipids in host defense against pathogens and their potential as therapeutic agents. Chem. Phys. Lipids.

[50] Fluhr J.W., Kao J., Jain M., Ahn S.K., Feingold K.R., Elias P.M. (2001). Generation of free fatty acids from phospholipids regulates stratum corneum acidification and integrity. J. Invest. Dermatol..

[51] Lee D.-Y., Huang C.-M., Nakatsuji T., Thiboutot D., Kang S.-A., Monestier M., Gallo R.L. (2009). Histone H4 is a major component of the antimicrobial action of human sebocytes. J. Invest. Dermatol..

[52] Desbois A.P., Gemmell C.G., Coote P.J. (2010). *In vivo* efficacy of the antimicrobial peptide ranalexin in combination with the endopeptidase lysostaphin against wound and systemic meticillin-resistant *Staphylococcus aureus* (MRSA) infections. Int. J. Antimicrob. Agents.

[53] Clinical and Laboratory Standards Institute (2008). Methods for Dilution Antimicrobial Susceptibility Tests for Bacteria That Grow Aerobically.

[54] Clinical and Laboratory Standards Institute (2001). Methods for Antimicrobial Susceptibility Testing of Anaerobic Bacteria.

[55] Isenberg H.D., American Society for Microbiology (1992). Synergism testing: Broth microdilution checkerboard and broth macrodilution methods. Clinical Microbiology Procedures Handbook.

